# Dental technician pneumoconiosis mimicking pulmonary tuberculosis: a case report

**DOI:** 10.1186/s12890-016-0293-2

**Published:** 2016-09-07

**Authors:** Han Loong Tan, Mohamed Faisal, Chun Ian Soo, Andrea Y. L. Ban, Roslina Abdul Manap, Tidi M. Hassan

**Affiliations:** Respiratory Unit, Department of Medicine, Universiti Kebangsaan Malaysia Medical Centre, Jalan Yaacob Latiff, Bandar Tun Razak, 56000 Cheras, Kuala Lumpur Malaysia

**Keywords:** Case report, Dental technician pneumoconiosis, Pulmonary tuberculosis

## Abstract

**Background:**

Dental laboratory technicians are at risk of developing occupational respiratory diseases due to exposure to various potentially toxic substances in their working environment. Since 1939, few cases of silicosis among dental technician have been reported.

**Case presentation:**

We illustrate a 38 year-old female, who worked in a dental laboratory for 20 years, initially treated as pulmonary tuberculosis and chronic necrotising aspergillosis without much improvement. Computed tomography guided lung biopsy and bronchoscopic transbronchial lung biopsy were performed. Lung tissue biopsies showed presence of refractile dental materials within the areas of histiocyte proliferation. The diagnosis of dental technician pneumoconiosis was obtained and our patient underwent pulmonary rehabilitation.

**Conclusions:**

This case highlights the importance of obtaining a detailed occupational history in tuberculosis endemic area, as pulmonary tuberculosis is a great mimicker of other respiratory diseases.

## Background

Dental technician pneumoconiosis has been described since the late 1930s. However, minerologic analysis of the presence of a new type of dust-induced fibrotic lung disease due to exposure to cobalt chromium molybdenum (CoCrMo) alloys which contain cobalt, chromium, molybdenum, and traces of other metals, such as silicon and manganese [1]. Therefore, dental technician pneumoconiosis is a complex pneumoconiosis or designated as mixed dust pneumoconiosis.

Pulmonary tuberculosis (PTB) is a great mimicker of many respiratory diseases and is endemic in Malaysia. We report a 38 year-old female, who worked in a dental laboratory for 20 years, initially treated as pulmonary tuberculosis and chronic necrotising aspergillosis without much improvement. A search for an alternative diagnosis which led to a lung biopsy revealing multiple black and brown coloured refractile dental materials led to the diagnosis of dental technician pneumoconiosis. To our knowledge, dental technician pneumoconiosis has not been reported in Malaysia and this could be the index case in our country. This case report highlights yet again, that although the threshold to treat smear-negative PTB is low in high TB incidence countries such as Malaysia, PTB mimics other chronic respiratory diseases that are preventable. As importantly, this case report emphasizes the need of a local epidemiologic study to determine the prevalence of dental technician pneumoconiosis and the implementation of necessary safety measures to prevent this avoidable disease.

## Case presentation

A 38-year-old non-smoker female with no known medical illness, presented with chronic cough for one year associated with intermittent low-grade fever, reduced appetite and significant loss of weight. The cough usually occurred in the morning with whitish sputum production. She also complained of a 2-day history of hemoptysis. She had no night sweats and there was no contact history with PTB patient. She denied shortness of breath.

**Fig. 1:**
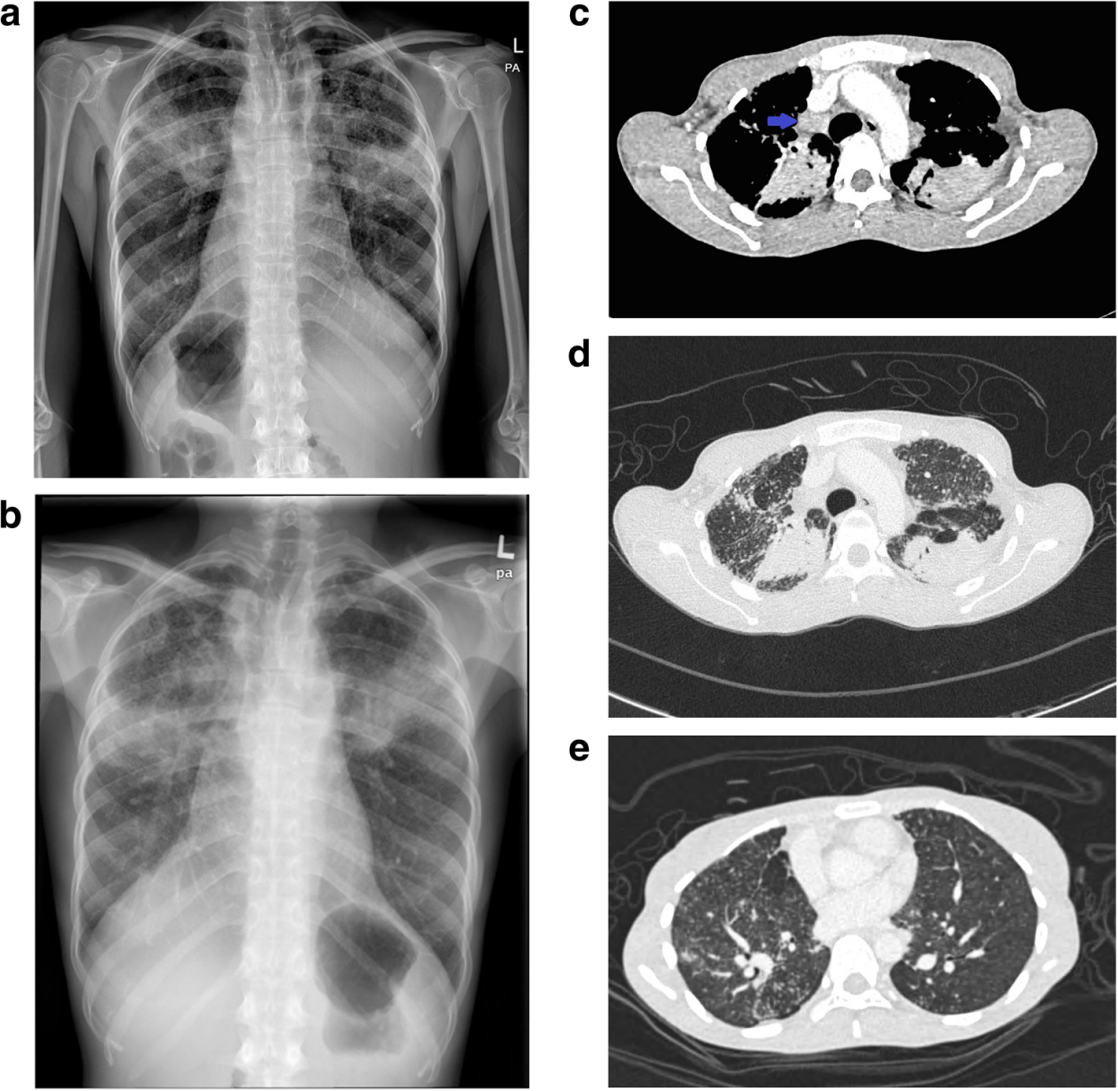
**a** Chest x-ray showed fibrotic changes at both upper zones with reticulo-nodular opacity over the upper and middle zone of both lungs. **b** Repeated chest x-ray after 54 doses of anti tuberculous medications showed absence of radiological improvement with similar reticulo-nodular opacity over bilateral lung field. **c** Mediastinal view of CT thorax showed enlarged right paratrachea lymphadenopathy (*Blue arrow*). **d** Consolidative changes with air bronchogram at the posterior segment of the left upper lobe and the apico-posterior segment of the right upper lobe. **e** The presence of lung nodules with tree in bud appearance

On examination, she appeared cachectic and had low-grade fever with temperature of 37.5 **°**C, blood pressure of 127/78 mmHg, pulse rate of 90 beats/min and oxygen saturation of 98 % under room air. The patient had no cervical lymphadenopathy. Chest auscultation revealed inspiratory crackles at bilateral upper and middle zones. She had leucocytosis with white cell count of 12.5 × 10^9^/L (neutrophil 77 %) and elevated erythrocyte sedimentation rate (72 mm/hour). Mantoux test, sputum acid-fast bacilli direct smear as well as mycobacterium culture and sensitivity were all negative. Her chest X-ray (CXR) (Fig. 1a) showed bilateral upper and middle zone reticulo-nodular opacity with a slight left-sided tracheal deviation. Pulmonary function test showed obstructive lung disease (FEV1/FVC of 68 %) with reduced FEV1 (0.61 l, 26 % of predicted) and FVC (0.90 l, 33 % of predicted).

As Tuberculosis (TB) is endemic in our country, she was initially treated as smear negative PTB based on the chronicity of her respiratory symptoms, and bilateral upper lobe reticulo-nodular opacity. However, after 54 doses of intensive anti-tuberculosis therapy, there was no improvement in respiratory symptoms or radiological changes (Fig. 1b). Computed tomography (CT) thorax (Fig. 1c–e) was subsequently performed revealing lung nodules with tree-in-bud appearance at bilateral upper, right middle and right lower lobes. Fibrotic changes were seen at the left apical region. There were enlarged right paratracheal and precarina lymph nodes with the largest node measuring 1.1 × 1.4 cm (precarina). Besides, there were consolidative changes with air bronchogram at both upper lobes, which suggested concomitant pneumonia. Bronchoscopy was performed and all segmental bronchus were narrowed with oedematous mucosa. Bacterial culture and mycobacterium polymerase chase reaction from BAL were negative but aspergillus antigen was positive (titre: 0.682), which raised the possibility of chronic necrotizing aspergillosis. Itraconazole 200 mg twice daily was added, in which she received for 12 weeks without much clinical improvement. The non-responsiveness to both anti-tuberculosis and anti-fungal therapy prompted a search for an alternative diagnosis. A more thorough occupational history revealed a significant exposure to hazardous dust containing quartz. She worked in a dental laboratory manufacturing dental prosthesis for 20 years with 54 working hours per week. She did not wear personal protective equipments when handling the investment material that contains quartz. In addition, her symptoms improved during her vacations.

**Fig. 2:**
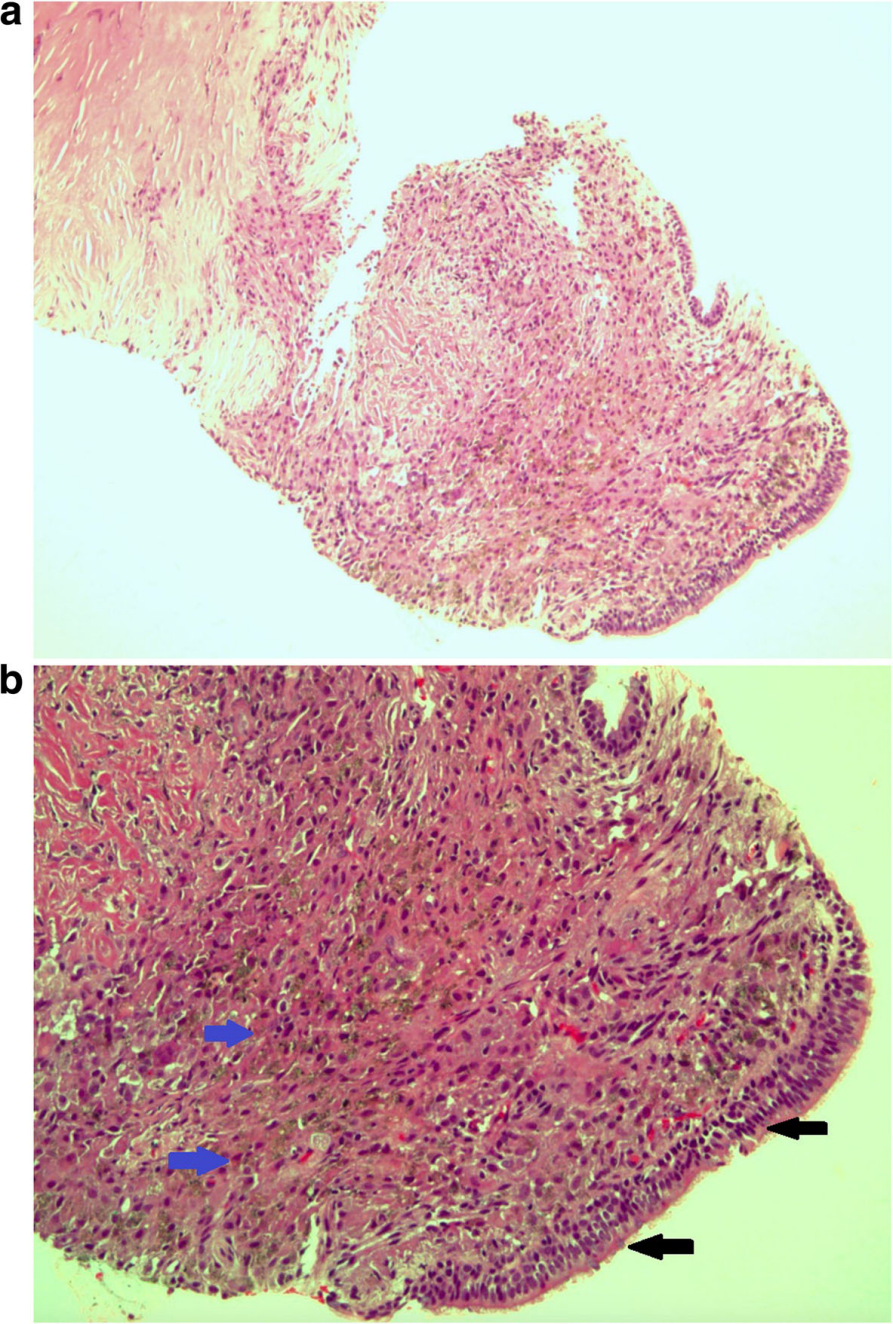
**a** and **b** CT guided lung biopsy. Haematoxylin and eosin stain revealed absence of normal alveolar tissue, which were replaced by fibrous tissue. There were areas of histiocytes proliferation with the presence of refractile dental material (*blue arrow*). The fibrous tissue was lined by ciliated tall columnar respiratory epithelium (*black arrow*)

She underwent a CT guided lung biopsy (Fig. 2a and b) over the consolidative area at right upper lobe, which showed absence of normal alveolar tissue, replaced by fibrous tissue. There were areas of histiocyte proliferation with multiple black and brown coloured refractile dental materials within. There were no multinucleated giant cell, caeseating granuloma or evidence of malignancy. A repeat bronchoscopy with transbronchial lung biopsy (TBLB), which was performed due to persistence of respiratory symptoms, revealed similar histological findings with presence of refractile dental materials.

Together with the findings from the CT thorax and histological examination of the lung biopsies, a diagnosis of dental technician pneumoconiosis was made. Despite explanation on the natural progression of the disease, she opted to continue working as a dental technician to support her family. Therefore, she was advised to use personal protective equipments during her working hours in the dental laboratory. Her respiratory symptoms, functional status and lung function capacity are being assessed and monitored during respiratory clinic follow-up. Currently, with on going pulmonary rehabilitation programme and proper usage of personal protective equipments, her cough symptoms reduce. Her effort tolerance and serial chest radiography every 4 to 6 months are stable.

Dental technician pneumoconiosis cases have been reported since 1939. Until the 1980s, minerologic analysis of early cases revealed presence of silicon, a new type of dust-induced fibrotic lung disease due to exposure to cobalt chromium molybdenum (CoCrMo) alloys which has been found among dental technicians manufacturing metal-framed dentures [1]. The prevalence of dental technician pneumoconiosis reported in literature ranges from 4.5 to 46 % [2–7] (Table 1). The vast difference in the prevalence among these studies could be due to difference in exposure time, level of metal dust in the dental laboratories, presence of exhaust ventilation and use of different radiological modalities in diagnosing pneumoconiosis.

**Table 1:** Prevalences of Dental Technician Pneumoconiosis

References	Year	Mean duration of work as a dental technician (years),	Participants	n, no of cases	Radiological diagnosis	Prevalence (no in 100 participants)
Rom et al. [2]	1984		178	8	CXR	4.5
Selden et al. [3]	1995	26.0	37	6	CXR	16.2
Doğan et al. [4]	2010		36	5	CXR	13.8
Berk et al. [5]	2014	14.0	32	8	CXR	25
Ergun et al. [6]	2014		893	90	CXR	10.1
Karaman et al. [7]	2014	16.7	76	35	HRCT Scan of Thorax	46.1

Prevalences of dental technician pneumoconiosis were reported as ranging from 4.5 to 25 % in chest radiograph-based studies. By using HRCT thorax, Karaman et al. reported presence of pneumocosiosis in 46.1 % of participants

Choudat et al. [8] found that the prevalence of dental technician pneumoconiosis was significantly higher (22.2 %) in dental technicians with exposure more than 30 years as compared to those with exposure less than 30 years (3.5 %, *p* < 0.004). Kahramanet al. [7] reported pneumoconiosis in almost half of the participants (46.1 %), a much higher prevalence compared with previous chest radiograph-based studies. High-resolution computed tomography (HRCT) scan of thorax has high sensitivity in detecting pneumoconiosis and is able to detect very early radiological changes.

Pulmonary tuberculosis is a great mimicker of many respiratory diseases and is endemic in Malaysia. In the year 2010, the local incidence of pulmonary tuberculosis was 81.4 per 100,000 populations and the numbers of new cases in the country continue to rise [9]. In contrast, dental technician pneumoconiosis is not commonly encountered. Thus, when this patient presented with chronic cough, constitutional symptoms and bilateral heterogenous consolidation on chest radiography predominantly at the upper lobe, an initial diagnosis of smear negative pulmonary tuberculosis was made. However, in view of the significant occupational exposure to hazardous dust containing quartz and lack of therapeutic response to anti-tuberculous medications, HRCT thorax and lung biopsy were performed to confirm a case of pneumoconiosis. The diagnosis of pneumoconiosis had improved the patient’s compliance to wear personal protective equipments when processing dental materials. This is an occupational hazard and preventive measures should be reinforced to avoid future incidence.

Cough, sputum production and dyspnoea are common respiratory symptoms in dental technician pneumoconiosis. Persistent coughing and expectoration were reported by 34 and 50 % of dental technicians respectively [7]. Nevertheless, respiratory symptoms were not useful in predicting presence of pneumoconiosis. Ergun et al. [6] observed that respiratory symptoms were more frequent among dental technicians with pneumoconiosis compared to those without. However, a significant correlation between the rate of pneumoconiosis and the presence of the respiratory symptoms were not observed in other studies [8, 10].

According to Berk et al. [5], parenchymal opacity was identified in 31 % by CXR and in 69 % by HRCT in 32 dental technicians. In a cross-sectional study of 76 dental technicians by Kahraman et al. [7], round opacities were the commonest HRCT thorax findings, followed by irregular/linear opacities, pleural abnormalities and emphysema. The majority of the round opacities were located at upper (28 %), middle (24 %) or both upper and middle zones (31 %). In our patient, large opacities were seen over both upper and middle zones without pleural thickening. Her CT thorax showed predominant lung nodules at both upper lobe and right middle lobes with consolidation and fibrotic changes. Unfortunately post-primary tuberculosis tends to involve these similar areas, and thus was the initial working diagnosis in this patient.

Previous studies had shown a deterioration of pulmonary function test in patients with dental technician pneumoconiosis. Radi et al. [11] investigated 134 dental technicians and 131 control subjects in six provinces of France. The mean value for FVC, FEF_25_ and FEF_50_ decreased significantly in male dental technicians. In other studies, respiratory function measures such as FEV1, FEV1/FVC and PEF were consistently lower in subjects with dental technician pneumoconiosis compared with those without pneumoconiosis, though the differences were not statistically significant [7, 8, 12]. Cigarette smoking, presence of emphysema and duration of occupational exposure had significant influence on patients’ pulmonary function test. Those who worked as dental technician for more than 15 years had significantly lower FEV1/FVC ratio. DLCO values were lower in dental technicians who smoke cigarettes and had emphysema [7]. Choudat et al. [8] observed a significant lower lung function among dental technicians than among control workers when occupational exposure and smoking habits were taken into account. Our patient had obstructive lung disease with decreased lung function parameters. Twenty years of occupational exposure to inorganic dust had resulted in significant lung parenchymal involvement in this patient.

By handling phosphate bonded investment materials that contain quartz, this patient could be at risk of silicosis. Normal alveolar tissue was replaced by fibrous tissue and there was histiocyte proliferation with presence of refractile dental material. Unfortunately, minerologic analysis was not performed in this case and identifying the exact inorganic dust causing pneumoconiosis may be difficult as dental technicians are exposed to a complex mixture of dust particles depending on the material used. Modern CoCrMo alloys used in the production of metal-framed partial dentures contain cobalt, chromium, molybdenum, and traces of other metals, such as silicon and manganese [1]. In reported cases of dental technician pneumoconiosis, lung biopsies were incinerated for mineralogical examination by scanning electron microscopy and energy dispersive x-ray spectrometer [13]. There were presence of silicon, phosphorus, calcium, iron, aluminium, chromium, cobalt and asbestos in the biopsied samples [13, 14]. Thus, dental technician pneumoconiosis is a complex pneumoconiosis or designated as mixed dust pneumoconiosis.

Continuous inorganic dust exposure may result in the progression of the disease. In a Turkish longitudinal study by Dogan et al. [15], 19 dental technicians were followed up for 7 years. After 7 years of occupational exposure in dental laboratories, the prevalence of pneumoconiosis increased from 13.8 to 47 %. It has been reported that even if the dust exposure ends, pneumoconiosis may progress. A periodic radiological and pulmonary function test survey in dental technicians with significant exposure to inorganic dust may allow early detection of pneumoconiosis. Monitoring inorganic dust level and installing local exhaust ventilation in dental laboratories could reduce the incidence of dental technician pneumoconiosis. In Sweden, the occupational exposure limit value for chromium, cobalt and quartz are 0.5, 0.05 and 0.1 mg/m^3^ respectively. Inorganic dust were generally below the permissible exposure limit in laboratories with exhaust ventilation, whereas in a dental laboratory without local exhaust ventilation, cobalt dust exceeded the Swedish occupational exposure limit by 32 times, while chromium and quartz dusts were also above the exposure limit [3].

Reporting this rare occupational lung disease could increase the awareness regarding the diagnosis, encourage an epidemiologic study to investigate its prevalence in our population and influence better occupational safety measures from both the employers and employees of the dental industry. Monitoring dust level in dental laboratories, installation of local exhaust ventilation and dust evacuation hood as well as usage of personal protective equipment by dental technicians are essential in preventing this occupational respiratory disease [16].

## Conclusions

Dental technician pneumoconiosis is a complex pneumoconiosis, which could be mistakenly diagnosed as pulmonary tuberculosis in TB endemic area. It is not commonly reported, as dental technicians who are exposed to various inorganic dusts may remain asymptomatic, which could lead to prolonged occupational exposure and an initial presentation at the advanced stage of the disease. Obtaining a detail occupational history is essential in diagnosing pneumoconiosis early, followed by an objective assessment with pulmonary function test, chest radiography, HRCT thorax and lung biopsy with mineralogical analysis. This is the index case of dental technician pneumoconiosis in our country and health-screening programmes should be conducted in all dental laboratories for the early prevention and/or diagnosis of pneumoconiosis.

## Abbreviations

BAL: Bronchoalveolar lavage
CoCrMo: Cobalt chromium molybdenum
CT: Computed tomography
CXR: Chest x-ray
HRCT: High resolution computed tomography
PTB: Pulmonary tuberculosis
TB: Tuberculosis
TBLB: Transbronchial lung biopsy
